# The Hidden Hand of Domestic Labor: Domestic Employers' Work Practices in Chicago, USA

**DOI:** 10.3389/fsoc.2019.00080

**Published:** 2019-12-11

**Authors:** Carolina Sternberg

**Affiliations:** Department of Latin American and Latino Studies, DePaul University, Chicago, IL, United States

**Keywords:** domestic workers, informal economy, employers, Latinx population, Chicago

## Abstract

An emergent body of scholarly work exists regarding the manifold dimensions and implications of domestic work, scholarship which draws from various standpoints and discipline traditions. Much existing literature deals specifically with the devaluation of domestic labor. A recent survey conducted in 14 metropolitan areas in the U.S. found that the domestic work industry is profoundly ethnocentric, gendered and racialized, with 23% of domestic workers earning below their state's mandated minimum wage. In 42 states, it is legal to pay domestic workers below minimum wage, since they are explicitly excluded from the protections of key federal labor laws and standards. In addition, many studies have repeatedly denounced the persistent gendered division of labor in the industry, and in particular have raised concerns about the disproportionate number of women of color in this occupation. Finally, given the private nature of domestic work and the unprotected conditions workers face, studies have pointed to the frequent hostile or even abusive relationships that employers have with their employees. Despite the wealth of research on domestic labor, relatively few studies conducted in the US have focused on the practices of domestic employers. There is also a dearth of research on domestic employment located specifically in the Midwest. The lacuna in this research motivated us to conduct a preliminary study on Midwestern employers' practices, in particular in Chicago and the surrounding suburbs. We argue that overlooking domestic employers' work practices prevents us from tackling the situations of abuse and disrespect that so frequently occur in this particular work environment.

## Introduction

An emergent body of scholarly work exists regarding the manifold dimensions and implications of domestic work, scholarship which draws from various standpoints and discipline traditions (Burnham and Theodore, 2012; Rosenbaum, 2017). Existing literature deals specifically with the devaluation of domestic labor. A relatively recent survey conducted in 14 metropolitan areas in the U.S. found that the domestic work industry is profoundly ethnocentric, gendered and racialized^1^, with 23% of domestic workers earning below their state's mandated minimum wage (Burnham and Theodore, 2012, p. 3–33). In 42 states, it is legal to pay domestic workers below minimum wage, since they are explicitly excluded from the protections of key federal labor laws and standards (e.g., bargaining laws, anti-discrimination laws, occupational safety and health, etc.). In addition, many studies have repeatedly denounced the persistent gendered division of labor in the industry, and in particular have raised concerns about the disproportionate number of women of color in this occupation (Metha, 2002; Hart, unpublished). Finally, given the private nature of domestic work and the unprotected conditions workers face, studies have pointed to the frequent hostile or even abusive relationships that employers have with their employees (Hondagneu-Sotelo, 2001, 2004; Hart, unpublished).

Despite the wealth of research on domestic labor, relatively few studies conducted in the US have focused on the practices of domestic employers (Tucker, 1987; Hondagneu-Sotelo, 1997; Young, 2010; Rosenbaum, 2014). In particular, these studies have pointed out that employers of domestic workers refuse to see themselves as employers and they have very little knowledge of, or rely on different rationalizations and sense of self-righteousness, to ignore state regulations governing their obligations as employers, e.g., paying federal taxes, social security, and medicare (Hondagneu-Sotelo, 1997; Young, 2010). Other studies have emphasized that domestic workers' working conditions are deeply shaped by a complex entanglement of racial relationships (Tucker, 1987; Burnham and Theodore, 2012). Following this last aspect, one of the authors notes that employers in Los Angeles characterize domestic workers as “forever foreign, intrinsically inassimilable (…)” (Rosenbaum, 2014, p. 138). Although the above studies have been remarkably influential, there is a dearth of research on domestic employment located specifically in the Midwest. The lacuna in this research motivated us to conduct a preliminary study on Midwestern employers' work practices. We argue that overlooking domestic employers' work practices prevents us from tackling the situations of abuse and disrespect that so frequently occur in this particular work environment.

Our current study explores domestic employers' work practices in Chicago and the surrounding suburbs. The research questions asked were later used to conduct a survey within DePaul community, the institution where am I currently employed. These questions were formulated upon drawing from the literature review discussed with my research assistant and from the information and handouts compiled from the US domestic employer network, *Hand in Hand* (Hand in Hand, 2019). The research questions were later discussed and slightly refined in one of my class sessions of my undergraduate course: “Domestic Workers Economy in the US and Beyond”, offered in January 2018. The following are the questions asked to the participants:

- What are the general demographics of domestic employers?
- What sorts of workers do domestic employers employ?
- What benefits and wages do employers offer their employees?
- What employment-related difficulties do employers encounter?
- What types of resources may be useful in fostering a better work environment for both the domestic employer and their employee(s)?

After collecting the results of the survey, students were also involved in discussing them and offering some conclusions that are included in this report.

## Methods

We decided to conduct this study using mixed methods in data collection, data analysis and interpretation of the evidence. Purposeful data integration enables researchers to seek a more panoramic view of their research landscape, viewing phenomena from different viewpoints and through diverse research lenses. Thus, this study uses quantitative data to explore domestic employers' work practices in Chicago and the surrounding suburbs. In addition, qualitative data were collected to gain insight into, (a) the different situations employers face when working with a domestic worker and, (b) reflect on their own practices.

We selected DePaul's staff and faculty, between 18 or older, as our sample for recruitment and who had been domestic employers within the past 5 years or were employing domestic help at the time the survey was administered. Research data collected in the survey and in the pre-screening (explained below) remained completely anonymous and the decision whether or not to be in the research did not affect participant's grades, status, or employment at DePaul University. For the purpose of this study we defined domestic employer as someone who is currently hiring or have hired someone in her/his home to clean, cook, assist, or care for members of the household within the past 5 years.

Before we distributed the survey on-line (from mid-November 2017 through late January 2018), we administered a pre-screening of DePaul faculty and staff. The pre-screening consisted of a very short email delivered to all DePaul faculty and staff (~2,600 employees) asking whether they were currently employing domestic help or they had done it sometime in the past 5 years. This allowed us to target directly the population that employs domestic help. Ultimately, the pre-screening survey identified 143 domestic employers on campus. Following the pre-screening, an email with a link to the actual survey was sent to the targeted population. In the end, we collected 63 surveys from mid-January to early February 2018. The information collected from the surveys was completely anonymous.

The surveys consisted of eight questions that asked participants about their common work practices as employers of domestic workers (e.g., type of worker, average hourly wage, existence of contract or written agreement between the employer and employee, benefits received, resources needed). We also collected some personal information about the participants, such as gender, age, income, and whether participants live in Chicago or Chicago suburbs to be able to identify some demographic patterns.

At the end of the survey we included a paragraph inviting respondents to participate in a focus group in order to share work experiences, identify positive and negative scenarios, and share best work practices among employers. We discuss this aspect further in the section “focus group analysis.”

## Analysis

Respondents were predominantly female (58.7%) and between 35 and 64 years old. Nearly 70% (69.8%, total 44) of the respondents reported having a household income above $100,000, while 13 decided not to answer this question. Respondents were also more likely to live in Chicago vs. the suburbs (50.6%).

### Types of Workers

Respondents identified a wide range of types of workers they employed in their household (Table 1). More than 34% of the respondents reported employing a housekeeper and nearly 13% indicated that they have employed a part-time childcare provider. Twelve respondents chose the “Other” response and specified a type of worker not listed on the survey. However, some of these respondents' answers were actually already included in the categories listed. For example, babysitters are included under the label “childcare provider.” All participants' responses are summarized below.

**Table 1:** Types of workers specified by survey and by respondent.

**Type of worker**	**Frequency**	**Percentage of sample**
**SPECIFIED BY SURVEY**		
Full-time childcare provider	5	7.9
Part-time childcare provider	8	12.7
Housekeeper	22	34.9
Full-time senior attendant	1	1.6
Part-time senior attendant	2	3.2
Full-time home attendant	0	0
Part-time home attendant	1	1.6
**SPECIFIED BY RESPONDENT**		
Au Pair	1	1.6
Cleaning lady/service	8	12.7
Dog-walker	1	1.6
Babysitter	2	3.2
Undisclosed	12	19

### Written Contracts and Benefits

We also assessed whether employers provided their employees with a contract and/or benefits as domestic employee compensation is inconsistent due to the unregulated nature of this work. Respondents indicated they offered highly variable wages to their employees, with benefits being even more variable. As illustrated in Table 2, the majority of employers who responded to this question did not provide employees with written contracts or benefits such as overtime pay, sick days, parental leave, or medical leave. However, in some instances benefits may not have been applicable to the employers' unique situations. For example, 36 respondents (57.1% of the sample) said they did not need to offer overtime pay to their employees, as their employees never worked more than 40 h per week. The chart below shows the frequency of respondents' indication that they *did* provide the benefit in question.

**Table 2:** Written contracts and benefits received by employees.

**Benefits**	**Frequency (employees providing the benefit)**	**Percentage**
Written contract/agreement	8	12.7
Overtime pay	2	3.2
Sick days	12	19
Parental leave	4	6.3
Medical leave	6	9.5
Undisclosed	31	49

### Wages

At the time the survey instrument was created, minimum wage in Chicago was $11.00/h. No standard minimum wage exists for the surrounding suburbs. More than half of the respondents reported paying their employees above the minimum wage in Chicago, with a full third of the sample paying nearly double the minimum wage (Table 3). Unfortunately, nearly another third of respondents did not respond to the question, which was the highest non-response rate of any question in the non-demographic portion of the survey.

**Table 3:** Hourly payment.

**Wage**	**Frequency**	**Percent**
<$11.00	2	3.2
$11–13	1	1.6
$14–16	8	12.7
$17–19	11	17.5
$20+	21	33.3
Undisclosed	20	31.7

### Difficulties in the Workplace

Respondents were asked to indicate whether they had experienced certain difficulties in offering benefits or living wages to their employees (Table 4). The survey offered a range of difficulties respondents could “check” that they had experienced, as well as an “other” option with a text entry box for respondents to indicate other difficulties not listed. Their responses are summarized below:

Of respondents who chose the “other” option, nine (60%) commented that they weren't sure what sorts of benefits or wages should be offered an employee who works for them only sporadically/occasionally, or that they did not believe benefits needed to be offered at all in such cases. This was especially true if the employee was contracted by an agency. Examples of such comments were:

- “I pay a service for light housekeeping on a biweekly basis. The service sometimes sends [sic] 2, 3, or 4 people to clean; I pay the same amount each time. **I do not know what the workers' hourly rate of pay is**.”
- “**The conversation and finances get complicated with overtime**, it's easier to agree on set hours (50 in my case) and a straight hourly rate. Hours above this are at employees [sic] discretion and at a different rate. I pay a living wage, but there's not enough flexibility in what the law prescribes.”
- In general, “I pay per job when in terms of housekeeping. [F]or the childcare I do pay more than a minimum wage.”
- “This is pay-per-visit work.”
- “We are less formal. Childcare providers end up getting 5 weeks+ off per year, but **we do not count the days like at an office job**. We always say yes when she needs time for medical, family, etc., and she does not abuse that.”

**Table 4:** Difficulties experienced in offering benefits or living wages to employees.

**Reason**	**Frequency**	**Percent**
I don't have enough information about what to do	2	3.2
It's uncomfortable to discuss these things with my employee	4	6.3
I can't afford to pay a living wage	3	4.8
My employee doesn't want an agreement/contract	9	14.3
Other	15	23.8
Undisclosed	30	48

### Resources for Employers

In the interest of providing domestic employers with resources to guide their employment habits, we asked respondents to select a list of resources that could be useful in navigating workplace situations. Respondents were also able to specify other resources they felt would be useful via text entry (Table 5).

**Table 5:** Resources for employers.

**Resource**	**Frequency**	**Percent**
Templates of working agreements	8	12.7
A conversation guide for difficult conversations	4	6.3
A guide about relevant state and federal laws	12	19.0
A checklist listing what you should ideally provide an employee	12	19.0
A report on the benefits others in your area provide employees	11	17.5
Other	6	9.5
Undisclosed	10	16

Of the six respondents who specified “other,” three indicated none of the above resources would be useful without providing other context. The other responses were as follows:

- “If I were to employ anyone full-time, I would want all of the above except ‘difficult conversations'.”
- “[N]one because the agency does this.”

Although the answers were succinct and did not provide a great deal of information, the first comment highlights the possibility that employers may be more open to resources if they employed full-time workers. The second comment suggests that employers who contract their workers from an agency may not need the same resources as those who find their workers elsewhere.

### Focus Group Analysis

After collecting the survey data, the next step in this study was to conduct a focus group to further understand domestic employers' work practices. This activity was created for employers to share work experiences when employing domestic workers, identify positive and negative scenarios, and share best work practices among them. In terms of our study, the focus group was geared toward developing a more thorough understanding of the situations employers face when working with a domestic worker and reflect on his or her own practices.

Unfortunately, recruiting DePaul employers for the focus group failed. The close relationship between my role as a researcher and the subject being studied, i.e., both employers and myself work in the same institution, may have discouraged many employers to reveal information they are not comfortable sharing in a focus group. We instead attended a 90 min-workshop on domestic employment organized by “Hand in Hand,” a US domestic employer network founded in 2010 by a group of domestic employers and their allies. This workshop had very similar purposes and objectives to the focus group we originally designed, given the fact that we closely followed the past experiences of Hand in Hand in developing workshops of this nature, topic and population. In any case, the Hand in Hand workshop was developed independently from our research and outside the DePaul community; however, we felt it was an interesting opportunity to supplement and integrate with our survey results and further advance our research. The facilitator of this workshop, a young woman in her early 30 s working as a teacher and caregiver was in charge of recruiting participants for this workshop. Roughly seven women in their 20–30 s attended the workshop, including the workshop facilitator. The majority of the participants were expecting a baby or already had young children at home.

#### Concerns Raised

Most of the concerns raised by attendees revolved around fair pay. Attendees repeatedly noted that they were unsure how to negotiate wages with employees and were also unaware of resources that could aid such conversations. Questions were also raised regarding overtime pay, vacation time, and sick days. The attendees stressed that they wanted to pay their employees fairly but were struggling to balance this desire with their complex financial situations.

Attendees also stated that they were unsure how to communicate with employees, both in terms of frequency of communication and the level of familiarity. One attendee stated that it was difficult for her to address concerns with her employee without sounding hostile or accusatory. Based on these remarks, the group explored the complexities of treating employees with warmth and friendliness while still maintaining a professional relationship. The workshop facilitator recalled an instance in which a domestic employer confessed to her, “Our employee is like family—so I hope she doesn't ask for a raise!” The facilitator used this example to show that becoming overly familiar with one's employee could lead to a lack of professionalism in the relationship, and further implied that it could result in wage theft or abuse.

#### Strategies Discussed

The workshop facilitator later shared strategies and ideas for resolving each issue. Guidance was offered regarding the following topics:

*a) Work agreements:* Employers and employees should work collaboratively crafting work agreements, with both parties agreeing to and initialing each portion. Additionally, it is helpful for the employer and employee to have a trial period after the initial contract is drafted; then, based on this trial period, the original contract may be revised with further clarifications.

*b) Fair pay:* Domestic workers should receive guaranteed income, even during periods in which an employer does not require the worker as often. For instance, if an employer stays at home for a week and does not require their worker's full-time help during that time, the employer must recognize that the employee still needs that week's income.

*c) Communication:* There should be transparency and regular communication between the employer and employee. Regular check-ins, even as often as once a week, are crucial to establishing trust.

## Final Remarks

We recognize that the sample size we used for our survey, 63 participants, was smaller than anticipated. However, as a reminder, this size was obtained after the pre-screening and the survey were administered. Given these constraints, we understand that the results obtained may be skewing the central observations offered below.

Scholars, activists, and practitioners who are involved in the industry of care work, would largely agree that the relationship between employers and employees is complex due to the nature of this type of work. Across the global North and South, domestic workers usually perform their work in a non-traditional workplace and behind closed doors, their work is widely unregulated, and has been historically devalued. Ultimately, all these aspects combined constitute strong limitations for improving their working conditions.

In the US, it was not until 2010 that domestic workers slowly began to gain more recognition and rights as a work force. The first Bill of Rights for Domestic Workers was passed in NY, followed by California, Hawaii, Connecticut, Oregon, Nevada, and Massachusetts. Recently, Illinois became the 8th state to pass a comprehensive Bill of Rights on January 1st, 2017 and the city of Seattle on July 23rd, 2018. This bill ensures that domestic workers receive minimum wage, protections against sexual harassment, and the right to 1 day off if they work for more than 20 h for an employer. However, it will take some time for this new legislation to be enforced.

Drawing on the literature presented above and our preliminary study based in Chicago, we suggest that employers need to understand that domestic work is real work, and be informed about domestic workers' rights, benefits and compensation. As stated previously, the stigmatized and unregulated nature of this work may render employers unaware of best work practices. Secondly, despite the fact that “domestic work is work” explicitly stated in the 2011 ILO convention no. 189, in many cases employers neither formally recognize domestic workers as legitimate “workers,” nor do they recognize themselves as employers (Hondagneu-Sotelo, 1997). Finally, there is a general perception among employers that he/she has special constitutional protections in a household setting compared to more traditional workplace settings where federal and state regulations do not fully apply (Young, 2010).

From our preliminary study, it follows that there seems to be no recognition on the part of either party that the employee is performing their duties in a professional workplace, even if it is also someone's home.

In other words, the legal and professional relationship between employer and employee becomes blurred because neither party recognizes the household as the worker's formal work environment. Thus, in order to establish a mutually constructive and beneficial relationship between employer and employee, and to ensure a good work environment and fair conditions for domestic workers, legitimizing this form of labor is essential.

## Successes and Challenges

We would like to thank the employers who participated in this study, the BRI fellowship and the Women's and Gender Studies Department (WGS) for supporting this research.

We recognize that the sample size we used for our survey, while adequate, was smaller than anticipated. In addition, employers were very selective in the type and extent of information they disclosed. We recognize the implications and bias of distributing a survey within our own university. The reasons that motivated us to do so were two-fold: (a) Faculty members had already conducted surveys to DePaul's faculty, students, and/or staff on diverse and sensitive topics; these suggested a diversity of interests, concerns and commitment to conducting critical research; (b) DePaul University is committed to promoting diversity, social justice and community engagement among its core values. Our study advocates for social justice in terms of bringing more awareness about best work practices among employers of domestic workers and helping promote public policies that bring respect to domestic workers in each and all of our communities.

Finally, we will consider other populations and ways for recruiting employers for a focus group outside of the university in the future.

## Scholarly Products Derived/Deriving From this Project

This study enhances existing literature on domestic labor, particularly with regards to domestic employers. Yet further research and action is needed to document the complexities of the employer-employee work relationship. This research should:

- Systematically examine and document the working relationship and practices that employers maintain with domestic workers.
- Share this information with workers' centers, activists, practitioners, and policy makers that work together to improve workers' protections and to reinforce recently available legislation on worker's protections.
- Share this information with employers, to be better informed about the type of work that domestic workers do as well as their rights, and to encourage them to follow best work practices.

## Data Availability Statement

The datasets generated for this study are available upon request to the corresponding author.

## Ethics Statement

The studies involving human participants were reviewed and approved by IRB DePaul University. The patients/participants provided their written informed consent to participate in this study.

## Author Contributions

CS contributed in the conception and design of this study and wrote and revised the entire manuscript.

### Conflict of Interest

The author declares that the research was conducted in the absence of any commercial or financial relationships that could be construed as a potential conflict of interest.

## Footnotes

^1^According to this study, for example, U.S. white nannies in 2011 earned 30% more than other racial/ethnic groups, in particular Latina.
